# Chloroplast Genome Variation and Evolutionary Analysis of *Olea europaea* L.

**DOI:** 10.3390/genes11080879

**Published:** 2020-08-03

**Authors:** Erli Niu, Chengying Jiang, Wei Wang, Yu Zhang, Shenlong Zhu

**Affiliations:** 1 Zhejiang Academy of Agricultural Sciences, Hangzhou 310021, China; niuerli@zaas.ac.cn (E.N.); wangw@zaas.ac.cn (W.W.); zhangy_lk@zaas.ac.cn (Y.Z.); 2 Gansu Academy of Forestry, Lanzhou 730020, China; jcytxb@126.com

**Keywords:** *Olea europaea* L., comparative chloroplast genome, genetic diversity, phylogenetic analyses

## Abstract

Olive (*Olea europaea* L.) is a very important woody tree and favored by consumers because of the fruit’s high-quality olive oil. Chloroplast genome analysis will provide insights into the chloroplast variation and genetic evolution of olives. The complete chloroplast genomes of three accessions (*O. europaea* subsp. *cuspidata* isolate Yunnan, *O. europaea* subsp. *europaea* var. sylvestris, and *O. europaea* subsp. *europaea* var. frantoio) were obtained by next-generation sequencing technology. A total of 133 coding regions were identified in the three chloroplast genomes without rearrangement. *O. europaea* subsp. *europaea* var. sylvestris and *O. europaea* subsp. *europaea* var. frantoio had the same sequences (155,886 bp), while *O. europaea* subsp. *cuspidata* isolate Yunnan (155,531 bp) presented a large gap between *rps16* and *trnQ-UUG* genes with six small gaps and fewer microsatellites. The whole chloroplast genomes of 11 *O. europaea* were divided into two main groups by a phylogenetic tree and *O. europaea* subsp. *cuspidata* formed a separate group (Cuspidata group) with the other subspecies (Mediterranean/North African group). Identification of consistency and diversity among *O. europaea* subspecies will benefit the exploration of domestication events and facilitate molecular-assisted breeding for *O. europaea*.

## 1. Introduction

Olive (*Olea europaea* L.) is a famous woody tree in the world and has been cultivated for about five to six thousand years in Mediterranean countries [1,2,3]. Except for a few fermented table olives, most olive fruits are used for oil extraction. Because of the mechanical method, olive oil is regularly consumed in its crude form without loss of nutrients. Therefore, it is considered as “liquid gold” and popular among consumers all over the world [4,5]. The olive belongs to the *O. europaea* species, which comprises of six subspecies, including *O. europaea* subsp. *europaea* (Mediterranean basin), *O. europaea* subsp. *maroccana* (Macaronesia), *O. europaea* subsp. *cerasiformis* (Macaronesia), *O. europaea* subsp. *guanchica* (Macaronesia), *O. europaea* subsp. *laperrinei* (Saharan mountains), and *O. europaea* subsp. *cuspidata* (from South Africa to South Asia) [3,6,7]. For *O. europaea* subsp. *europaea*, the cultivated olive (*O. europaea* subsp. *europaea* var. *europaea*) and wild olive (*O. europaea*. subsp. *europaea* var. sylvestris) are differentiated. There are currently more than 2600 cultivars grown for oil extraction after a long period of domestication with biogeographic conditions and human influence [8]. Olive trees are primarily distributed in Spain, Italy, and Greece, where they enjoy the moderate temperatures and semi-arid Mediterranean climate. Nowadays, olive trees have been introduced into about 40 countries such as China, Australia, and the US [9].

Until now, more than 2000 olive accessions have been collected in the Olea databases (http://www.oleadb.it). The phenomenon of synonyms, homonyms, and unclear genetic relationship still exists among olive germplasms [10,11]. Researchers have done lots of studies on the molecular markers to distinguish different olive accessions, such as the amplified fragment length polymorphism (AFLP), simple sequence repeat (SSR), and single nucleotide polymorphism (SNP) [12,13,14]. D’Agostino et al. [15] and Zhu et al. [16] conducted whole-genome level SNP exploration for 97 and 57 olive cultivars, respectively. The two studies produced high identity-by-state values between different pairs of cultivars, which had formerly been considered the same cultivar in the past years. In addition, the screening of core loci provided a more efficient and faster method for identification of different olive germplasms [16,17]. Until now, the genomic sequencing of three olive trees, *O. europaea* subsp. *europaea* cv. leccino, *O. europaea* subsp. *europaea* cv. farga, and *O. europaea* subsp. *europaea* var. sylvestris, were available [18,19,20]. More studies identifying germplasm resources at the whole-genome level and determining the mechanism of agronomic traits need to be done urgently.

Organelle DNA genomes mtDNA and cpDNA are maternally inherited and provide scientists simple and fast methods to study the different genetic backgrounds of olive germplasms [21]. Molecular markers and organelle DNA sequences are available in olive. Using lengths of restriction fragments markers, Amane et al. [22] classified the chloroplast of 72 cultivars and 101 wild olives into five chlorotypes and found that the same chlorotype was predominant over the whole geographical distributions of cultivated olive and the oleaster forms. More numerous variant chlorotypes were observed in oleasters than in cultivated olive, although they all displayed low variation [22]. With PCR-RFLP and microsatellite markers, 143 cultivated olive, 334 wild olive, 77 subspecies, and 1 outgroup (*Olea woodiana* Knobl.) were classified into five clades with only 15 chlorotypes [23]. Mariotti et al. [24] and Besnard et al. [21] conducted chloroplast DNA sequencing and found that the sizes of olive chloroplast DNA varied from 155,531 to 155,896 bp with low nucleotide divergence (<0.07%) among the lineages. Olive trees shared a high similarity in the *europaea* subspecies with more variation between different subspecies [21]. Here, we sequenced the cpDNAs of *O. europaea* subsp. *cuspidata* isolate Yunnan, *O. europaea* subsp. *europaea* var. sylvestris, which displayed significant differences from most olive cultivars in tree characteristics, fruit traits, and resistance. As a control, the cultivated olive *O. europaea* subsp. *europaea* var. frantoio was also employed to analyze genome variation and genetic association among olive chloroplasts. Through the analysis of structure comparison and evolution relation among all the *O. europaea* species, this study provides a better understanding of chloroplast variation and genetic evolution of olive at the whole-genome level.

## 2. Materials and Methods

### 2.1. Plant Material and DNA Extraction

Three olive accessions were collected and analyzed in this study including *O. europaea* subsp. *europaea* var. frantoio, *O. europaea* subsp. *europaea* var. sylvestris, and *O. europaea* subsp. *cuspidata* isolate Yunnan. The first two accessions were collected from Italy and Spain, respectively, while *O. europaea* subsp. *cuspidata* isolate Yunnan was collected from China. Fresh young leaves (~100 mg) were sampled from the new shoots and frozen in liquid nitrogen for further analysis.

Total DNA was isolated with modified cetyltrimethylammonium bromide (CTAB) method as described by Murray et al. [25]. Agarose gel electrophoresis (1.2%) was used to detect DNA integrity, purity, and concentration, and a qubit fluorometer was used to determine DNA concentration.

### 2.2. Sequencing and Data Quality Control

Complete DNA sequencing was done using Illumina’s next-generation sequencing technology. The genome sequencing was performed on the Illumina MiSeq 2000 (Illumina Inc., San Diego, CA, USA) with paired-end methods (150 bp). The raw sequence reads were filtered using the NGSQC Tool Kit v2.3.3 as follows: (1) remove adapter sequence in the reads; (2) remove the reads whose 5’-end base was unknown; (3) remove the reads with the quality value ≤ Q20; (4) remove reads whose unknown bases ≥ 10%; (5) remove reads whose length was less than 50 bp.

### 2.3. Chloroplast Genome Assembly and Annotation

The quality of the raw reads was assessed by FastQC [26] and carried out by Cutadapt [27]. Clean reads were assembled into scaffolds using the de novo assembler SPAdes [28] and further assembled using Blastn and exonerated with *O. europaea* subsp. *europaea* var. manzanilla (FN996972.1) as a reference. Sequence extension, hole filling, and splicing were performed with paired-read iterative contig extension (PRICE) and MITObim (https://github.com/chrishah/MITObim). The chloroplast genes were annotated using the DOGMA and UGENE ORFs finder tool [29] and visualized with OGDraw 1.2 [30].

Each of the assembled cpDNA sequences has been submitted to GenBank and acquired the following accession numbers: MT182984 and MT182986 for *O. europaea* subsp. *europaea* var. frantoio and *O. europaea* subsp. *europaea* var. sylvestris, and MT182985 for *O. europaea* subsp. *cuspidata* isolate Yunnan.

### 2.4. Comparative Genomic and Repetitive Sequences Analysis

Except for the three *O. europaea* chloroplast genomes sequenced here, the other three subspecies genomes, including *O. europaea* subsp. *laperrinei* (MG255765.1), *O. europaea* subsp. *guanchica* (MG255764.1), *O. europaea* subsp. *maroccana* (FN998900.2), were used for comparative genomic analysis. Sequence identity and rearrangement were performed using the mVISTA program with LAGAN mode [31] and Mauve alignment [32].

Repetitive simple sequence repeat (SSR) sequences were analyzed with MISA software (http://pgrc.ipk-gatersleben.de/misa/). Four types of repeat sequences, including forward, reverse, complement, and palindrome sequences, were determined by REPuter software with a minimum repeat size of 20 bp as described by Liu et al. [33].

### 2.5. Phylogenetic Analysis

All of the nucleic acid sequences of 11 *O. europaea* chloroplast genomes were used to conduct the phylogenetic tree with *Olea lancea* (NC_042278.1) as the outgroup. In addition to the three *O. europaea* chloroplast genomes sequenced here, the other eight genomes included *O. europaea* subsp. *laperrinei* (MG255765.1), *O. europaea* subsp. *guanchica* (MG255764.1), *O. europaea* subsp. *maroccana* (FN998900.2), *O. europaea* subsp. *europaea* var. bianchera (NC_013707.2), *O. europaea* subsp. *europaea* var. manzanilla (FN996972.1), *O. europaea* subsp. *cuspidata* isolate Maui 1 (FN650747.2), *O. europaea* subsp. *cuspidata* isolate Guangzhou 1 (FN996944.1), and *O. europaea* subsp. *cuspidata* isolate Almihwit 5.1 (FN996943.2). These were obtained from the National Center for Biotechnology Information (https://www.ncbi.nlm.nih.gov). MAFFT 7.427 (https://www.ebi.ac.uk/Tools/msa/mafft/) and Gblocks (–t = d, –b5 = h) were used for multi-sequence alignment and editing. The phylogenetic tree was built using IQTREE 1.6.10 software (http://www.iqtree.org) with maximum likelihood method (GTR + I + G) and edited with Figtree 1.4.3 software (http://tree.bio.ed.ac.uk/software/figtree/).

## 3. Results

### 3.1. Assembly and Validation of Chloroplast Genome

**Figure 1:**
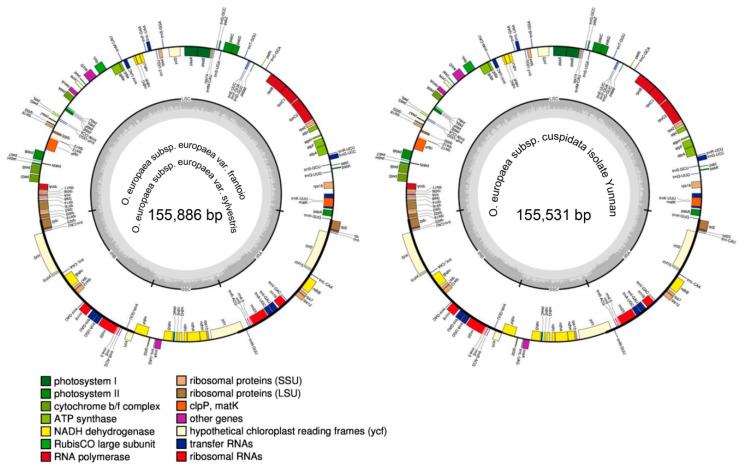
Chloroplast gene maps of *Olea europaea* subsp. *europaea* var. frantoio, *O. europaea* subsp. *europaea* var. sylvestris, and *O. europaea* subsp. *cuspidata* isolate Yunnan. Genes with different functions were shown in different colors. Those transcribed clockwise or counter-clockwise were shown inside or outside the circle. LSC, large single-copy region; SSC, small single-copy region; IR, inverted repeat.

The chloroplast genome sizes of *O. europaea* subsp. *europaea* var. frantoio, *O. europaea* subsp. *europaea* var. sylvestris, and *O. europaea* subsp. *cuspidata* isolate Yunnan were 155,886, 155,886, and 155,531 bp with 42512X, 35953X, and 48376X depth, respectively. After performing the de novo and reference-guided assembly with minor modifications, the complete chloroplast genome sequences of three *O. europaea* accessions were obtained (Figure 1). *O. europaea* subsp. *europaea* var. frantoio and *O. europaea* subsp. *europaea* var. sylvestris shared the completely same sequence (Figure 1; Table 1), which was consistent with *O. europaea* subsp. *europaea* var. manzanilla (FN996972.1) [21]. The genomes of all of the three *O. europaea* had two copies of inverted repeat (IR, 25,742 and 25,731 bp) separated by large single-copy (LSC, 86,611 and 86,279 bp) and small single-copy (SSC, 17,791 and 17,790 bp) regions (Table 1). There were 133 coding regions, including 88 protein-coding genes, 8 rRNA, and 37 tRNA (Figure 1; Table 2).

### 3.2. Genetic Structure of the Chloroplast Genome of Olive

**Figure 2:**
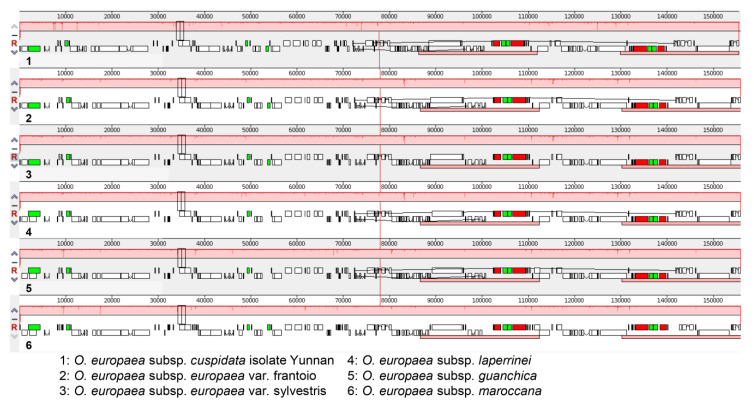
Synteny comparisons of six *O. europaea* chloroplast genomes. The chloroplast genome of *O. europaea* subsp. *europaea* var. sylvestris was used as reference sequence. Within each of the alignments, local collinear blocks were marked by the same color and connected by lines.

**Figure 3:**
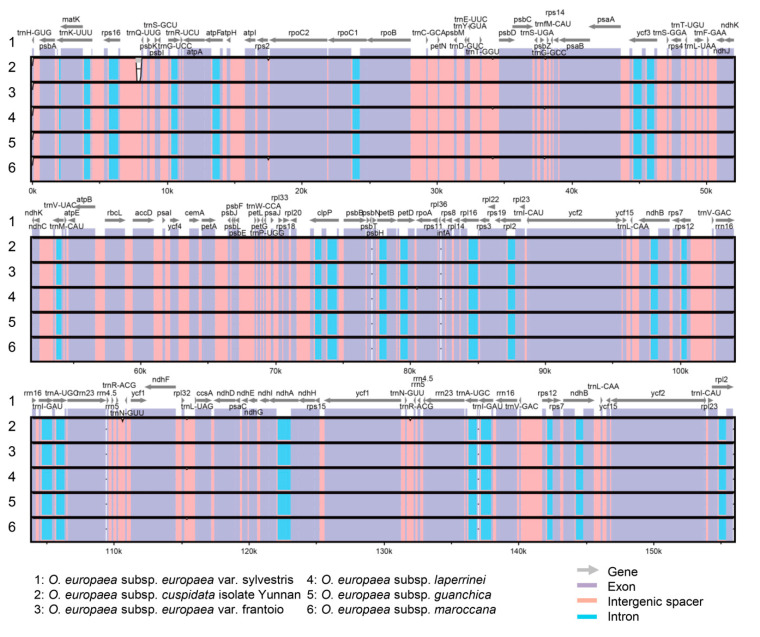
Comparisons of six *O. europaea* chloroplast genomes. Chloroplast genome of *O. europaea* subsp. *europaea* var. sylvestris was used as reference sequence, and the horizontal axis indicated the coordinates with other chloroplast genomes. Gene, exon, intron, and intergenic spacer were colored.

To make a comprehensive comparison of *O. europaea* chloroplast genomes, the gene structures of *O. europaea* subsp. *europaea* var. frantoio, *O. europaea* subsp. *europaea* var. sylvestris, and *O. europaea* subsp. *cuspidata* isolate Yunnan were drafted with the other three *O. europaea* subspecies, including *O. europaea* subsp. *laperrinei* (MG255765.1), *O. europaea* subsp. *guanchica* (MG255764.1), and *O. europaea* subsp. *maroccana* (FN998900.2) obtained from NCBI (https://www.ncbi.nlm.nih.gov). The six *O. europaea* chloroplast genomes showed collinear gene organization with no rearrangement that occurred (Figure 2). Compared to *O. europaea* subsp. *europaea* var. sylvestris, *O. europaea* subsp. *cuspidata* isolate Yunnan had a large gap between *rps16* and *trnQ-UUG* genes with six small gaps located in intergenic spacers (Figure 3). Furthermore, *O. europaea* subsp. *laperrinei*, *O. europaea* subsp. *guanchica,* and *O. europaea* subsp. *maroccana* had 4, 0, and 4 gaps, respectively.

### 3.3. IR Expansion and Contraction

**Figure 4:**
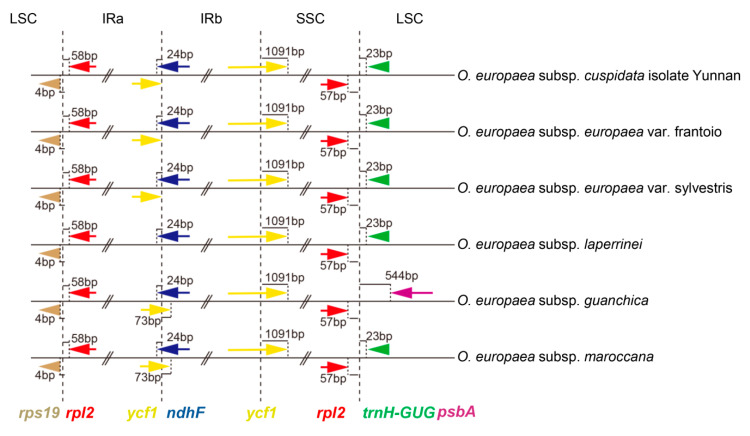
Border comparisons of six *O. europaea* chloroplast genomes. Chloroplast genome of *O. europaea* subsp. *europaea* var. sylvestris was used as reference sequence. LSC, large single-copy region; SSC, small single-copy region; IR, inverted repeat.

There were two significant differences of the chloroplast genomes among these six *O. europaea* accessions. *O. europaea* subsp. *laperrinei* and *O. europaea* subsp. *guanchica* lacked the ycf1 and the *trnH*-*GUG* gene near the IRa-SSC border and IRb-LSC border, respectively (Figure 4). While the nucleic acid sequences at the corresponding genes in these two samples were not significantly different from the other samples except for some SNPs, it was speculated that the ycf1 and the *trnH*-*GUG* genes were exhaustively annotated and existed.

We also found that the ycf1 gene at the boundary between IRa and SSC had different expansion and contraction. As in Figure 4, the *ycf1* gene from the three samples (*O. europaea* subsp. *cuspidata* isolate Yunnan, *O. europaea* subsp. *europaea* var. frantoio, and *O. europaea* subsp. *europaea* var. sylvestris) were right at the border of IRa and SSC, while in *O. europaea* subsp. *guanchica* and *O. europaea* subsp. *maroccana*, the *ycf1* gene was located across both IRa and SSC regions.

### 3.4. Repetitive Sequences and Hotspot Regions in Chloroplast Genomes

**Figure 5:**
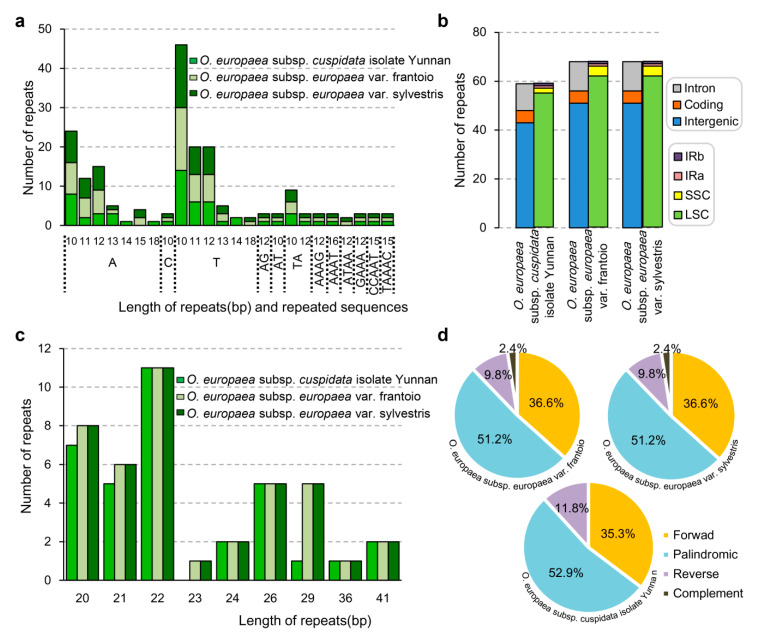
Statistical information of simple sequence repeats (SSRs) detected in *O. europaea* subsp. *europaea* var. frantoio, *O. europaea* subsp. *europaea* var. sylvestris, and *O. europaea* subsp. *cuspidata* isolate Yunnan. (**a**) Distribution of SSRs in the different regions; (**b**) length and repeated sequences; (**c**) type of SSRs with 20 bp or longer; (**d**) statistics of SSRs with 20 bp or longer.

To further explore more differences, the microsatellites of three *O. europaea* chloroplast genomes were also studied. There were 68, 68, and 59 microsatellites identified in *O. europaea* subsp. *europaea* var. frantoio, *O. europaea* subsp. *europaea* var. sylvestris, and *O. europaea* subsp. *cuspidata* isolate Yunnan, respectively (Figure 5a). For the 68 microsatellites identified from *O. europaea* subsp. *europaea* var. frantoio and *O. europaea* subsp. *europaea* var. sylvestris, 56 were mono-nucleotide, 6 were di-nucleotide, 4 were tetra-nucleotide, 2 were penta-nucleotide. No tri-nucleotide or hexa-nucleotide was found (Figure 5a). Among these microsatellites, 51, 5, and 12 microsatellites were located in the intergenic, protein-coding, and intron regions (Figure 5b). Of the 59 microsatellites identified from the *O. europaea* subsp. *cuspidata* isolate Yunnan, 48 were mono-nucleotide, 6 were di-nucleotide, 3 were tetra-nucleotide, and 2 were penta-nucleotide. No tri-nucleotide or hexa-nucleotide was found (Figure 5a). Among these microsatellites, 43, 5, and 11 microsatellites were located in the intergenic, protein-coding, and intron regions (Figure 5b).

For microsatellites with 20 bp or longer, 41, 41, and 34 repeats were detected from *O. europaea* subsp. *europaea* var. frantoio, *O. europaea* subsp. *europaea* var. sylvestris, and *O. europaea* subsp. *cuspidata* isolate Yunnan, respectively (Figure 5c). In detail, 20, 21, 22, 24, 26, 29, 36, and 41 bp-long repeats occurred in all of these three chloroplast genomes, while 23 bp-long repeats were only detected in *O. europaea* subsp. *europaea* var. frantoio, *O. europaea* subsp. *europaea* var. sylvestris. There were 51.2% and 52.9% considered as palindromic repeats in *O. europaea* subsp. *europaea* and *O. europaea* subsp. *cuspidata* isolate Yunnan (Figure 5d). No complement repeats were identified in *O. europaea* subsp. *cuspidata* isolate Yunnan (Figure 5d).

### 3.5. Genetic Phylogenetic Analysis

**Figure 6:**
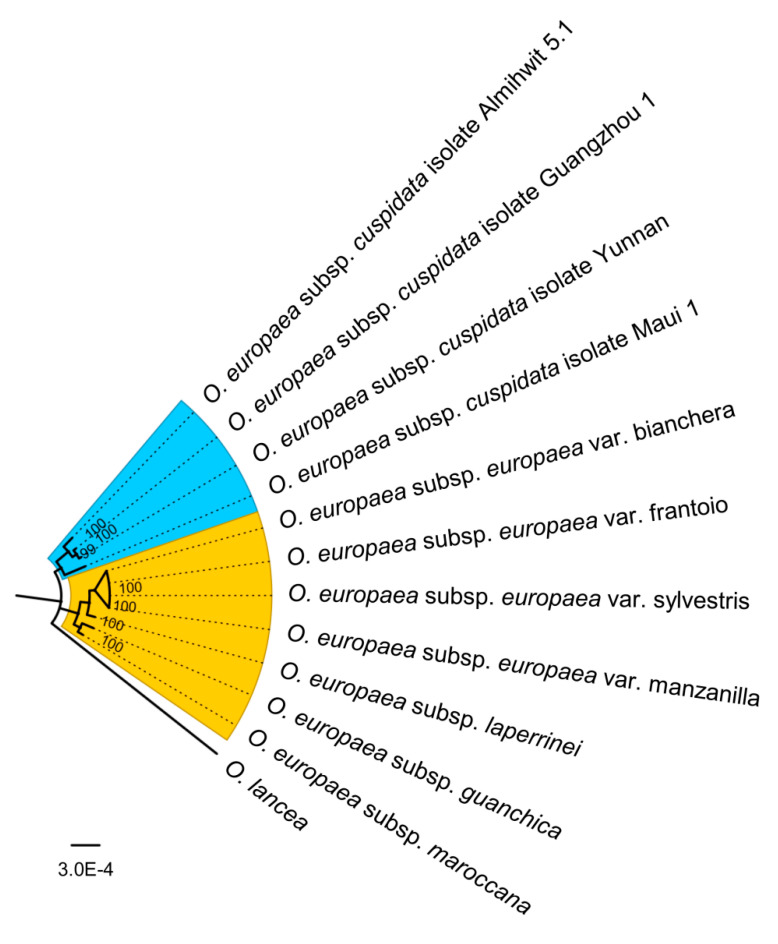
Phylogenetic analysis of *O. europaea* species. Whole chloroplast sequences of 11 *O. europaea* including *O. europaea* subsp. *europaea* var. frantoio (MT182984), *O. europaea* subsp. *europaea* var. sylvestris (MT182986), *O. europaea* subsp. *cuspidata* isolate Yunnan (MT182985), *O. europaea* subsp. *laperrinei* (MG255765.1), *O. europaea* subsp. *guanchica* (MG255764.1), *O. europaea* subsp. *maroccana* (FN998900.2), *O. europaea* subsp. *europaea* var. bianchera (NC_013707.2), *O. europaea* subsp. *europaea* var. manzanilla (FN996972.1), *O. europaea* subsp. *cuspidata* isolate Maui 1 (FN650747.2), *O. europaea* subsp. *cuspidata* isolate Guangzhou 1 (FN996944.1), *O. europaea* subsp. *cuspidata* isolate Almihwit 5.1 (FN996943.2), and *Olea lancea* (NC_042278.1) were used as the outgroup. Phylogenetic tree was built using IQTREE 1.6.10 software (http://www.iqtree.org) with maximum likelihood method (GTR + I + G).

Due to the low genetic diversity, the whole chloroplast genome sequences of 11 *O. europaea* were constructed the genetic phylogenetic analysis based on maximum likelihood method with *Olea lancea* (NC_042278.1) as the outgroup (Figure 6). *O. europaea* chloroplast genomes were classified into two branches. *O. europaea* subsp. *cuspidata* was relatively different from the rest and grouped as an individual branch, forming the cuspidata clade as Besnard et al. [23] described. Four *O. europaea* subspecies *O. europaea* subsp. *europaea,* including the wild and cultivated, *O. europaea* subsp. *laperrine*, *O. europaea* subsp. *guanchica,* and *O. europaea* subsp. *maroccana,* showed closer relationships and formed the Mediterranean/North African clade.

## 4. Discussion

Six olive subspecies are recognized as before [3,6,7]. Among them, *O. europaea* subsp. *europaea* is generally considered to include two differentiated variants: The cultivated (*O. europaea* subsp. *europaea* var. europaea) and wild (*O. europaea* subsp. *europaea* var. sylvestris) olive [34,35]. The two variants show overlapping distributions in the Mediterranean basin. Although the diversity of morphology and stress physiology is clear, the botanical and genetic studies have verified that the cultivated variants are derived from wild olives [34,35,36,37]. Single or multiple independent domestication events has been a debate [38]. Here, the chloroplast genome of *O. europaea* subsp. *europaea* var. sylvestris was first sequenced and showed exactly the same as *O. europaea* subsp. *europaea* var. frantoio. They also displayed a high similarity with cultivated olives, indicating that few differentiation events were present in *O. europaea* subsp. *europaea* chloroplasts. More exploration of domestication events should be conducted to study the genome sequences.

The phylogenetic analysis based on the whole chloroplast sequences showed that *O. europaea* occupied two main groups, the Mediterranean/North African and the Cuspidata groups, which confirmed previous research using polymorphic sites [23,24,39]. The genetic structure and repetitive sequences displayed the divergence clearly between *cuspidata* and other subspecies. Although *O. europaea* subsp. *cuspidata* isolate Yunnan had the same number of coding regions without rearrangement, a large gap exists between *rps16* and *trnQ*-*UUG* with six small gaps was present. Moreover, 59 microsatellites were identified from *O. europaea* subsp. *cuspidata* isolate Yunnan, compared to 68 found in *O. europaea* subsp. *europaea*. The results indicate high diversity between Cuspidata and Mediterranean/North African groups and further benefit the development of molecular markers.

In the genus *Olea*, only the cultivars of *O. europaea* are economically valuable, and *O. europaea* shows low genetic variation and obvious regionalization. *O. europaea* subsp. *cuspidata* has no economic value other than as an ornamental. The diversity of *O. europaea* subsp. *europaea* with other subspecies identified here could be used as an important gene resource to broaden the genetic background of olive cultivars through conventional or molecular breeding methods. They appear to be compatible using the conventional breeding methods. Ma et al. [40,41] reported that the variety Jinyefoxilan, derived from a cross between of *O. europaea* subsp. *europaea* var. frantoio and *O. europaea* subsp. *cuspidata* isolate Yunnan, had stronger abiotic stress-resistance tolerance, more vigorous vegetative growth, and a later flowering stage compared to the female parent. Our findings will provide more information on *O. europaea* subsp. *cuspidata* isolate Yunnan for molecular assisted breeding.

## Figures and Tables

**Table 1:** Summary of the three chloroplast genomes sequenced in this study.

Category	*O. europaea* subsp. *europaea* var. frantoio/*O. europaea* subsp. *europaea* var. sylvestris	*O. europaea* subsp. *cuspidata* isolate Yunnan
Total length	155,886 bp	155,531 bp
Length of large single copy (LSC) region	86,611 bp	86,279 bp
Length of small single copy (SSC) region	17,791 bp	17,790 bp
Length of inverted repeat (IR) region	25,742 bp	25,731 bp
GC content	37.8%	37.8%
Total number of genes	133	133
Number of protein encoding genes	87	87
Number of rRNA genes	8	8
Number of tRNA genes	37	37
Loci of JLA	86,612 bp	86,280 bp
Loci of JSA	112,353 bp	112,010 bp
Loci of JSB	130,145 bp	129,801 bp
Loci of JLB	155,886 bp	155,531 bp

**Table 2:** Genes identified in the chloroplast genome of olive.

Category for Genes	Group of Genes	Name of Genes
Self-replication	tRNA genes	*rrn4.5*, *rrn5*, *rrn16*, *rrn23*
	rRNA genes	*trnA*-*UGC*^†^, *trnC*-*GCA*, *trnD*-*GUC*, *trnE*-*UUC*, *trnF*-*GAA*, *trnfM*-*CAU*, *trnG*-*GCC*, *trnG*-*UCC*^†^, *trnH*-*GUG*, *trnI*-*CAU*, *trnI*-*GAU*^†^, *trnK*-*UUU*^†^, *trnL*-*CAA*, *trnL*-*UAA*^†^, *trnL*-*UAG*, *trnM*-*CAU*, *trnN*-*GUU*, *trnP*-*UGG*, *trnQ*-*UUG*, *trnR*-*ACG*, *trnR*-*UCU*, *trnS*-*GCU*, *trnS*-*GGA*, *trnS*-*UGA*, *trnT*-*GGU*, *trnT*-*UGU*, *trnV*-*GAC*, *trnV*-*UAC*^†^, *trnW*-*CCA*, *trnY*-*GUA*
	Small subunit of ribosome	*rps2*, *rps3*, *rps4*, *rps7*, *rps8*, *rps11*, *rps12*^§^, *rps14*, *rps15*, *rps16*^†^, *rps18*, *rps19*
	Large subunit of ribosome	*rpl2*^†^, *rpl14*, *rpl16*^†^, *rpl20*, *rpl22*, *rpl23*, *rpl32*, *rpl33*, *rpl36*
	DNA dependent RNA polymerase	*rpoA*, *rpoB*, *rpoC1*^†^, *rpoC2*
Genes for photosynthesis	Subunits of NADH-dehydrogenase	*ndhA*^†^, *ndhB*^†^, *ndhC*, *ndhD*, *ndhE*, *ndhF*, *ndhG*, *ndhH*, *ndhI*, *ndhJ*, *ndhK*
Genes for photosynthesis	Subunits of NADH-dehydrogenase	*ndhA*^†^, *ndhB*^†^, *ndhC*, *ndhD*, *ndhE*, *ndhF*, *ndhG*, *ndhH*, *ndhI*, *ndhJ*, *ndhK*
	Subunits of photosystem I	*psaA*, *psaB*, *psaC*, *psaI*, *psaJ*
	Subunits of photosystem II	*psbA*, *psbB*, *psbC*, *psbD*, *psbE*, *psbF*, *psbH*, *psbI*, *psbJ*, *psbK*, *psbL*, *psbM*, *psbN*, *psbT*, *psbZ*
	Subunits of cytochrome b/f complex	*petA*^†^, *petB*^†^, *petD*, *petG*, *petL*, *petN*
	Subunits of ATP synthase	*atpA*, *atpB*, *atpE*, *atpF*^†^, *atpH*, *atpI*
	Large subunit of rubisco	*rbcL*
Other genes	Maturase	*matK*
	Protease	*clpP* ^‡^
	Envelope membrane protein	*cemA*
	Subunit of acetyl-CoA-carboxylase	*accD*
	C-type cytochrome synthesis gene	*ccsA*
	Translational initiation factor 1	*infA*
Genes of unknown function		*ycf1*, *ycf2*, *ycf3*^‡^, *ycf4*, *ycf15*

^†^ Genes contain one intron; ^‡^ genes contain two introns; ^§^ genes that need trans-splicing.
